# Supplementary material for: The Role of T-Cadherin (CDH13) in Treatment Options with Garcinol in Melanoma
Source: Cancers (Basel). 2024 May 12;16(10):1853. doi: 10.3390/cancers16101853 (PMC11119778; doi:10.3390/cancers16101853)
Supplement: Supplementary file 1 [file cancers-16-01853-s001.zip › cancers-2995420-figures.pdf]

Supplementary Figure S1

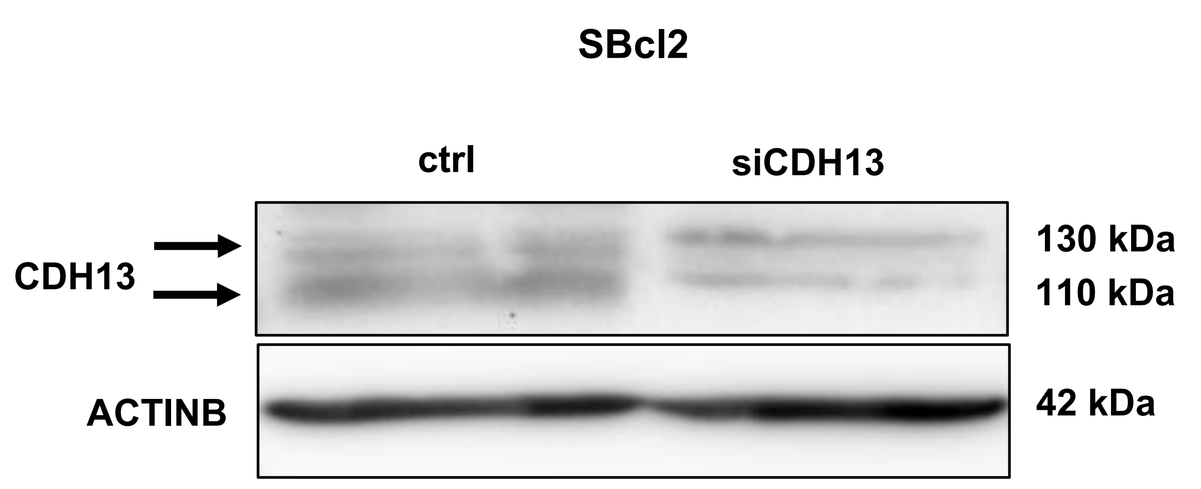

Supplementary Figure S1: Representative Western Blot images depicting protein expression level of CDH13. Equal loading was controlled by ACTINB (42 kDA) primary antibody. Cells were transfected with siCDH and siCTR, respectively, for 72 hours and total protein was extracted with subsequent Western Blot analysis.

Supplementary Figure S2

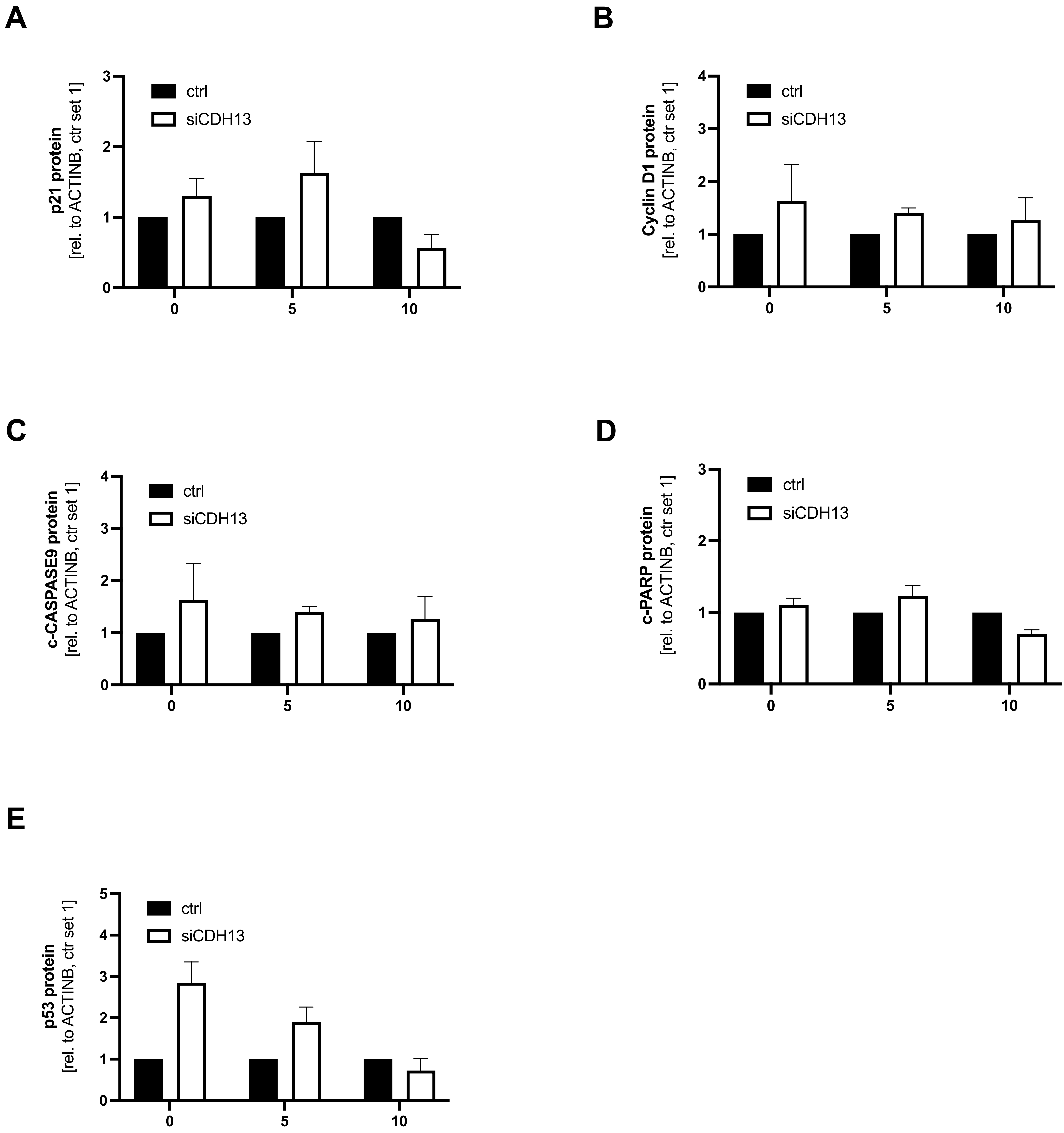

Supplementary Figure S2: A,B,C,D,E: Densitometry for Figure 4 of p21 (**A**), Cyclin D1 (**B)**, c-CASPASE9 (**C)**, c-PARP (**D**) and p53 (**E**) of SBcl2 cells transfected with siCDH13 and siCTR, respectively, and subsequently stimulated with garcinol for additional 24 h.
