# Supplementary material for: The Role of T-Cadherin (CDH13) in Treatment Options with Garcinol in Melanoma
Source: Cancers (Basel). 2024 May 12;16(10):1853. doi: 10.3390/cancers16101853 (PMC11119778; doi:10.3390/cancers16101853)

Figure 2 A

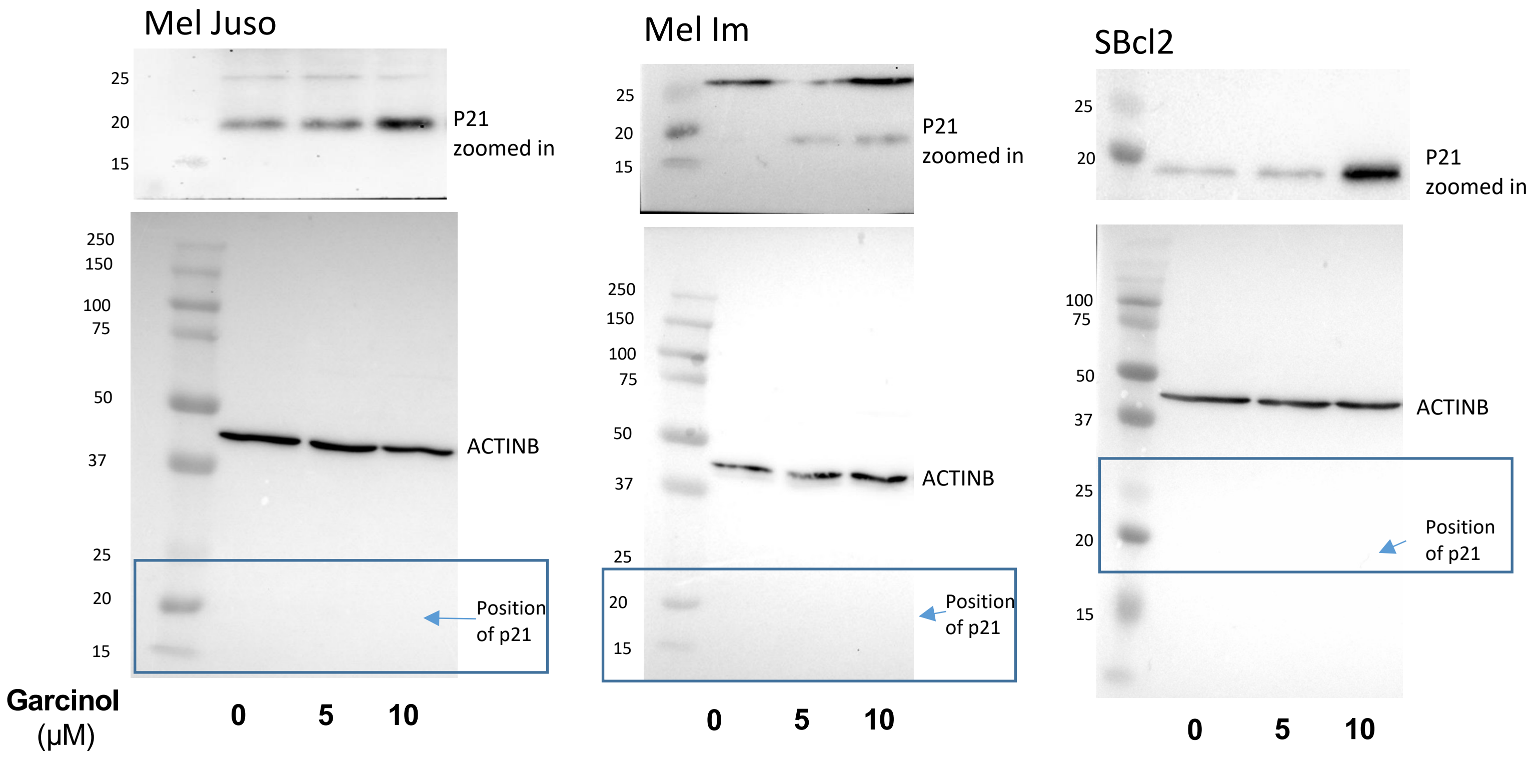

We do not have the complete western blots for p21 because the molecule was so difficult to detect at the edge of the gel. However, based on the comparison of the standard, we can see where the band is located and that it is the appropriate loading control.

Figure 2 C

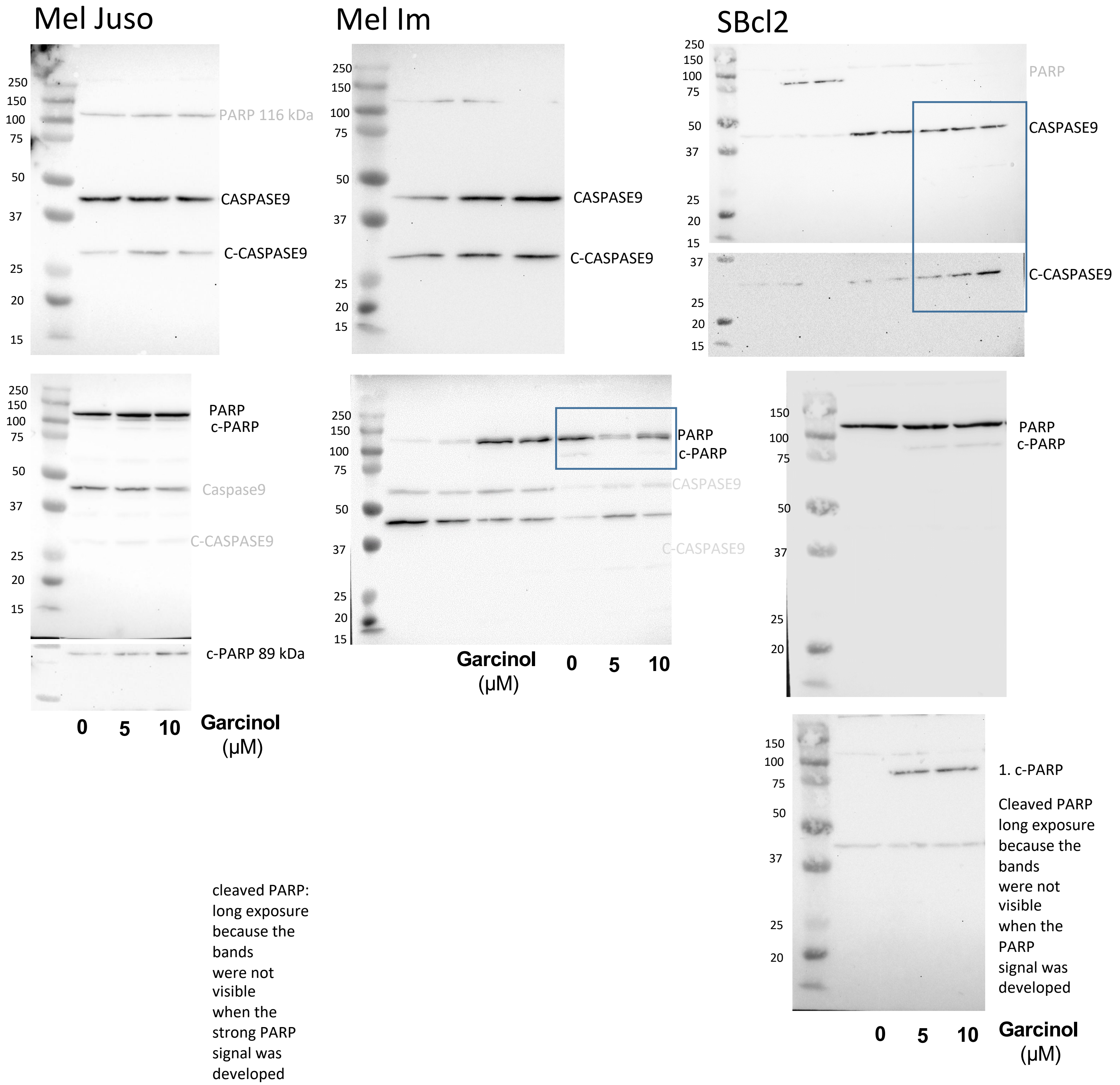
Mel Juso
Mel Im
SBcl2
250
150
100
75
50
37
25
20
15
PARP 116 kDa
CASPASE9
C-CASPASE9
PARP
c-PARP
Caspase9
C-CASPASE9
c-PARP 89 kDa
0
5
10
Garcinol
(μM)
PARP
CASPASE9
C-CASPASE9
PARP
c-PARP
CASPASE9
C-CASPASE9
Garcinol
(μM)
0
5
10
PARP
CASPASE9
C-CASPASE9
PARP
c-PARP
1. c-PARP
Cleaved PARP long exposure because the bands were not visible when the PARP signal was developed
0
5
10
Garcinol
(μM)
cleaved PARP: long exposure because the bands were not visible when the strong PARP signal was developed

Figure 3A

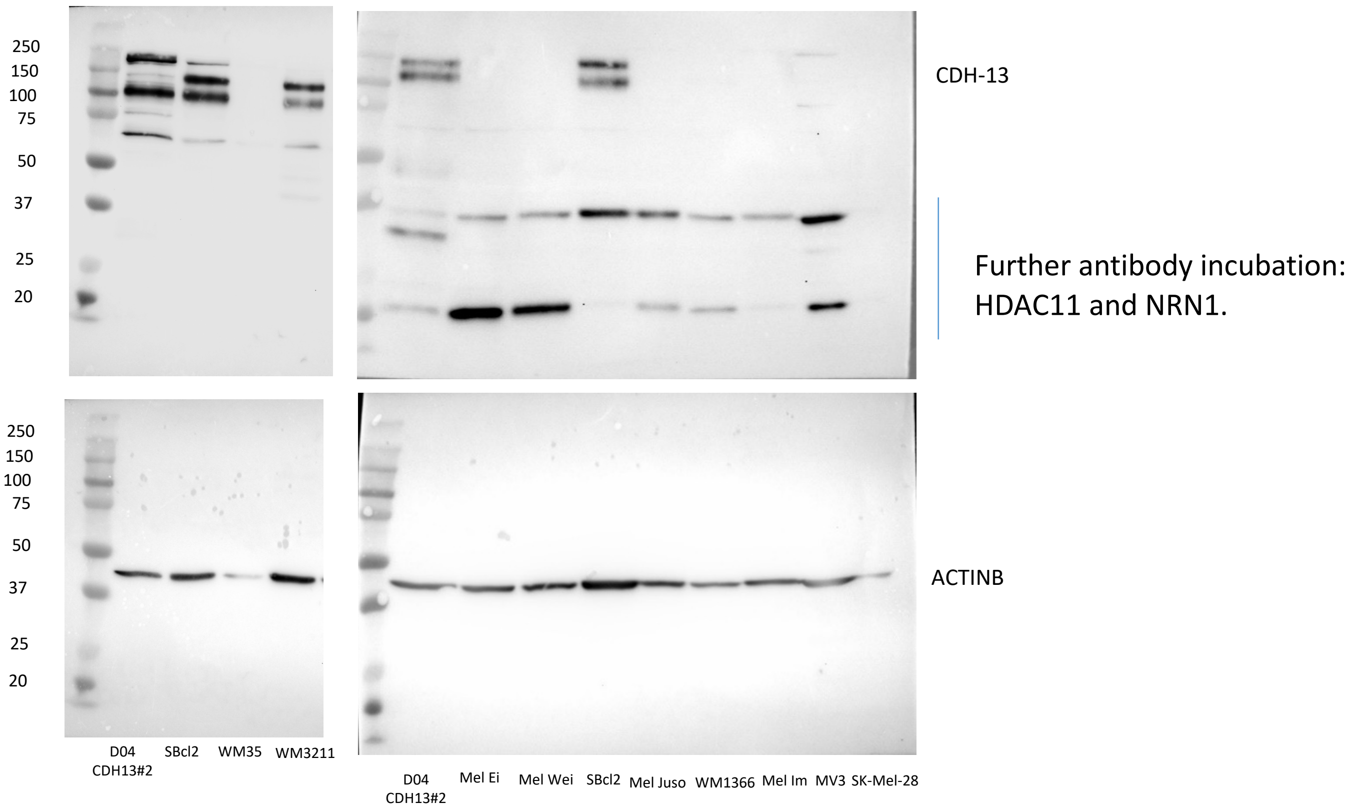

The double band observed in the western blots reflects the expression of the mature CDH13 (105 kDa) and the uncleaved CDH13 precursor (130 kDa). Lower bands are shedded CDH13.

Figure 3 B

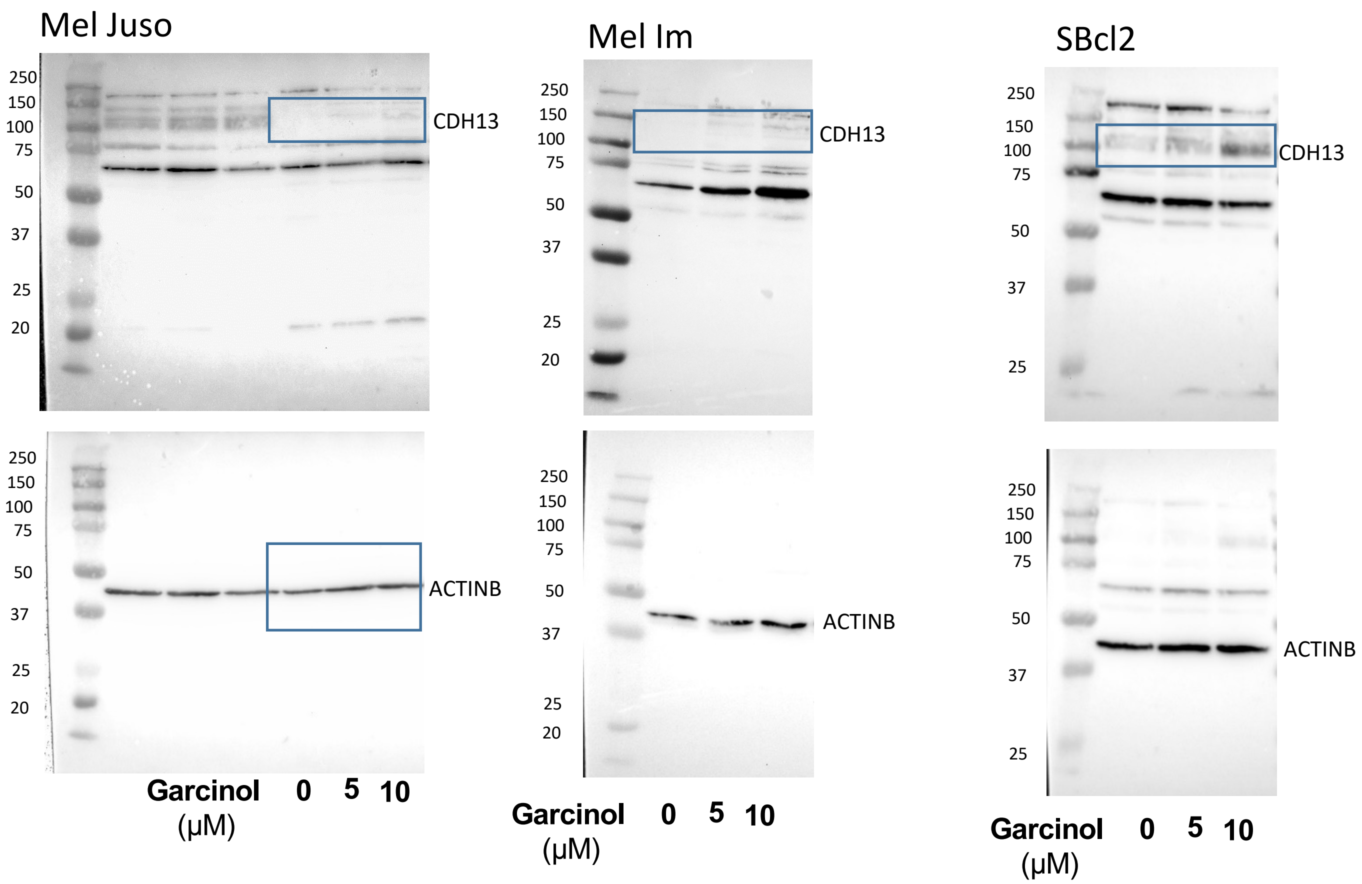

Mel Juso
250
150
100
75
50
37
25
20
CDH13
ACTINB
Garcinol (μM) 0 5 10
Mel Im
CDH13
ACTINB
Garcinol (μM) 0 5 10
SBcl2
CDH13
ACTINB
Garcinol (μM) 0 5 10

Figure 4 A, B, D, E, F

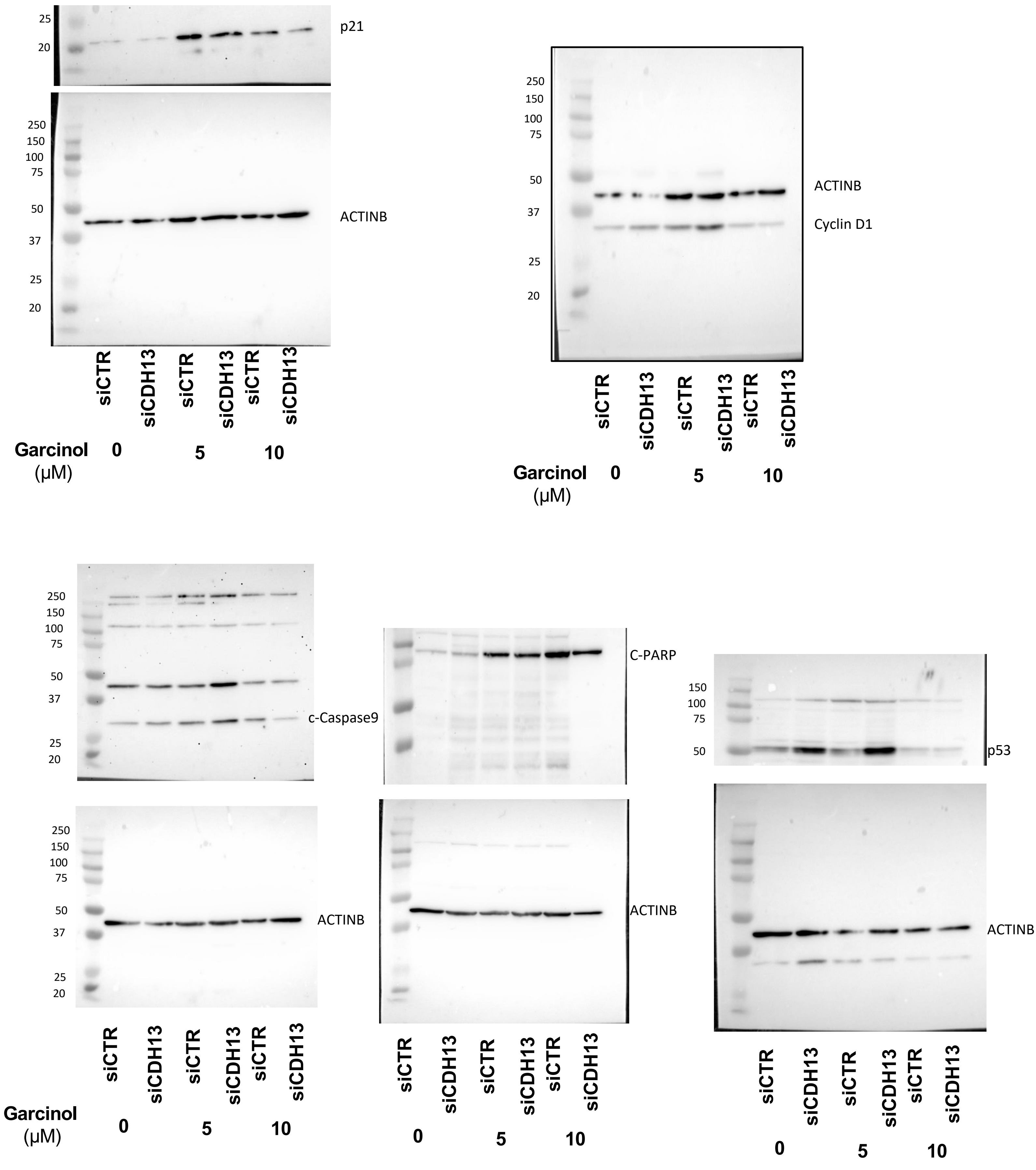

Supplementary Figure 1

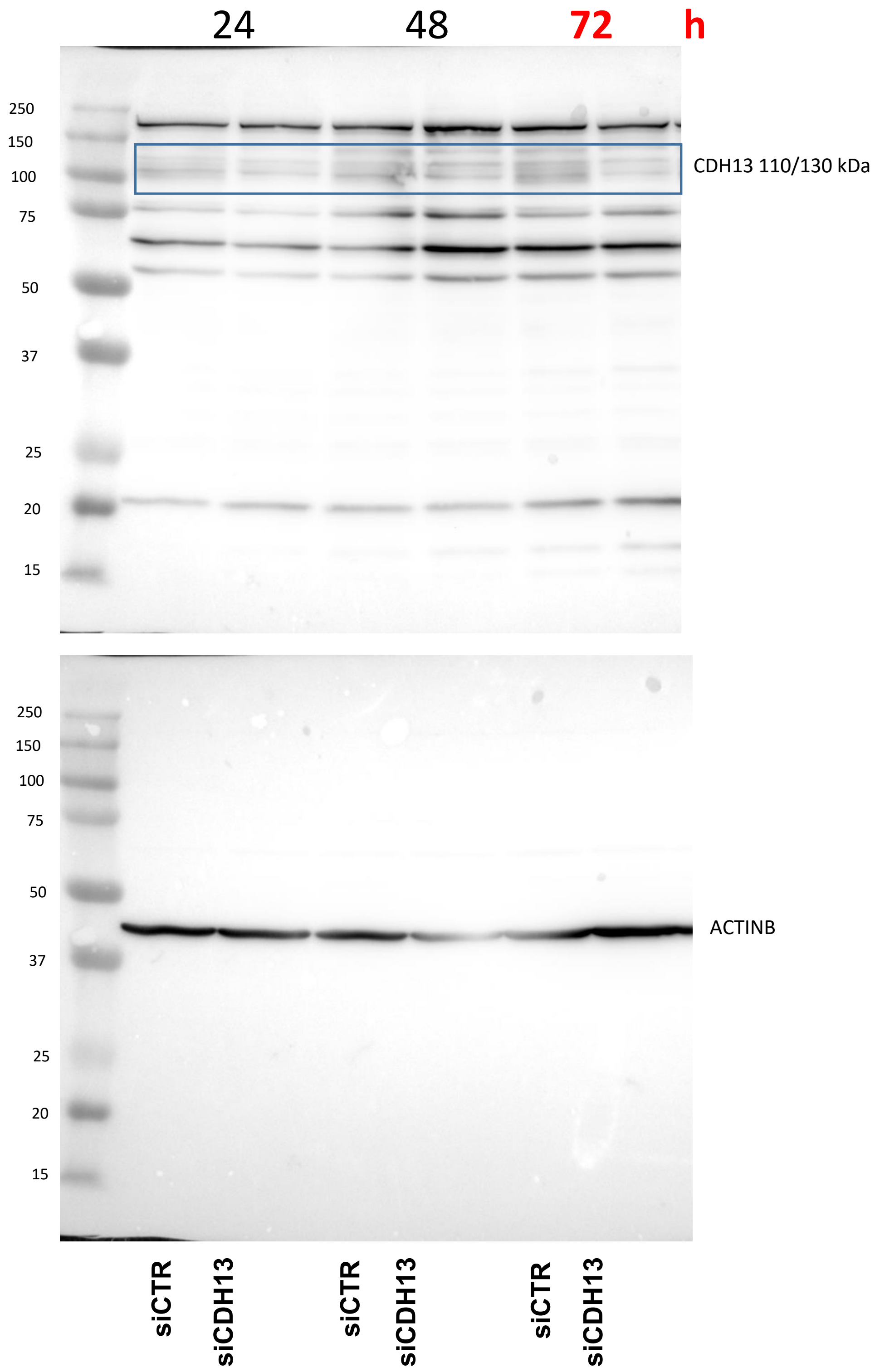

Supplement: Supplementary file 1 [file cancers-16-01853-s001.zip › cancers-2995420-file S1.pdf]
